# Central Pancreatectomy: Significance of Vascularity on Anastomotic Integrity and a Note on Reconstructive Technique

**DOI:** 10.7759/cureus.18617

**Published:** 2021-10-08

**Authors:** Suraj Girish, Nagaraj Kapil, Naveen Kannan

**Affiliations:** 1 General Surgery, Meenakshi Medical College and Research Institute, Kanchipuram, IND; 2 Surgical Gastroenterology, Meenakshi Medical College and Research Institute, Kanchipuram, IND; 3 General Surgery, Meenakshi Medical College Hospital and Research Institute, Kanchipuram, IND

**Keywords:** solid pseudopapillary neoplasm, octreotide, pancreaticojejunostomy, pancreaticogastrostomy, central pancreatectomy

## Abstract

Central pancreatectomy is a pancreatic parenchymal sparing surgery usually indicated for benign and borderline malignant tumors of the neck and proximal body of the pancreas. Due to the presence of extensive intra-pancreatic spread, pancreatic parenchyma sparing procedures such as central pancreatectomy are invariably deferred in pancreatic malignancy. The need for management of two pancreatic stumps with a usually soft texture and non-dilated ducts, given the indications, increases the risk of pancreatic fistula and therefore morbidity. Proximal stump management is usually a closure either by suture or stapler with reinforcements; the technique preferred depends on the experience of the surgeon and is mostly extrapolated from distal pancreatectomy. Distal stump management is the Achilles’ heel owing to the texture of the pancreas and pancreatic duct size. Need for additional mobilization may have a bearing on the perfusion of the pancreatic stump and hence may lead to clinically relevant leaks. The use of octreotide accentuating the said vascular insufficiency may not be an overstatement. Here we present a case of solid pseudopapillary tumor (SPT) of the neck and proximal body of the pancreas in which a central pancreatectomy with falciform patch closure of the proximal stump and binding pancreaticogastrostomy (PG) was contemplated and further we discuss the types of reconstruction with special reference to the vascular pattern of distal pancreas.

## Introduction

Central pancreatectomy is a procedure where the neck and a variable length of the body of the pancreas are resected. Alternative terminology has been “middle” or “median” pancreatectomy. Initially described for malignant tumors of the body, later widely adopted to treat benign and borderline malignant diseases of the pancreas such as intraductal papillary mucinous neoplasm (IPMN), serous cystic neoplasm (SCN), mucinous cystic neoplasm (MCN), and solid pseudopapillary neoplasm (SPN). Enucleation is another less invasive alternative for smaller similar lesions in which the likelihood of injury to the main pancreatic duct is negligible [1]. Conventional procedures such as extended pancreaticoduodenectomy and distal pancreatectomy with or without splenectomy are employed for malignant lesions in the neck and proximal body region in view of the higher rate of margin positive resections in spite of adequate macroscopic clearance due to intrapancreatic spread of pancreatic cancer cells [2]. Conventional procedures tend to resect a significant amount of normal pancreatic tissue leading to variable exocrine and endocrine insufficiency. On the other hand, central pancreatectomy preserves pancreatic parenchyma, but requires handling of two pancreatic remnants and is therefore reported to have a higher incidence of postoperative pancreatic fistula with a subsequent rise in morbidity and length of hospital stay [3]. The closure of the proximal stump can be achieved by sutures, staplers, stump anastomosis, or a combination of both sutures and staplers. Also reinforcements with a falciform patch, omental patch have been found to have variable benefits [4]. Reconstruction of the distal stump is of utmost importance as the risk of the postoperative pancreatic fistula is higher owing to the soft texture and the smaller caliber of the main pancreatic duct [5]. Herewith, we present a case of a solid pseudopapillary tumor (SPT) of the neck and body of the pancreas wherein a central pancreatectomy and binding pancreaticogastrostomy (PG) with trans-pancreatic U sutures were done along with falciform patch reinforced suture closure of the proximal stump.

## Case presentation

**Figure 1:**
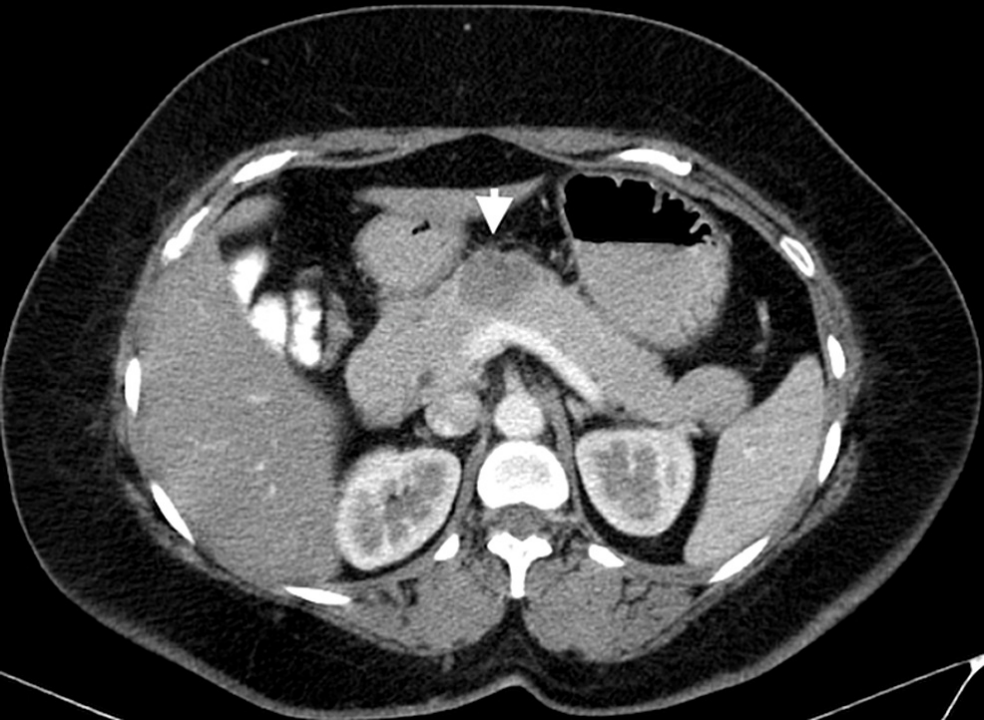
Contrast-enhanced CT abdomen image in portal venous phase showing a heterogeneously enhancing lesion in the neck and body of pancreas with normal main pancreatic duct and patent splenic vein.

A 35-year-old female presented with an incisional hernia. She had undergone laparoscopic surgery for a benign ovarian cyst two years ago. She was evaluated and incidentally found to have a cystic lesion in the neck and proximal body of the pancreas. She had a contrast-enhanced computed tomography (CECT) of the abdomen which showed a heterogeneously enhancing lesion measuring approximately 3.8 x 3.1 cm with few cystic foci and peripheral calcifications arising from the body of pancreas. The pancreatic duct appeared normal, with no obvious communication with the same (Figure 1). Based on imaging, she was suspected to have an MCN.

**Figure 2:**
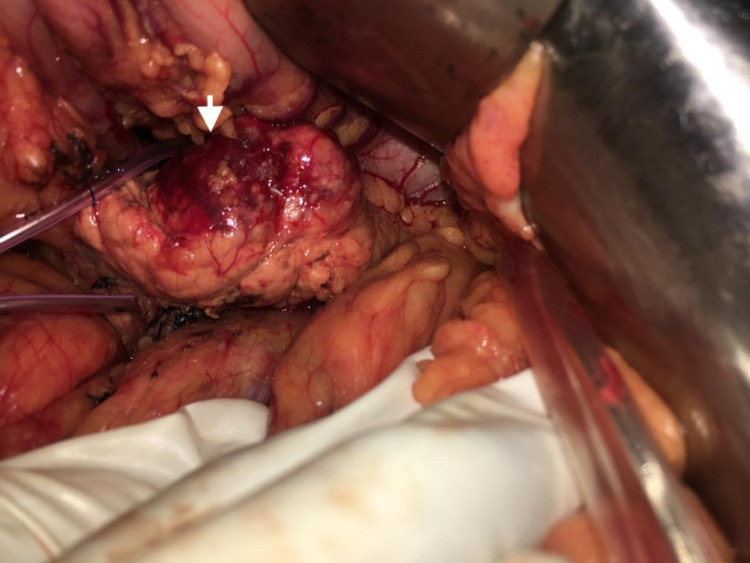
Intraoperative image of the lesion in the neck and body of the pancreas. 5Fr Infant feeding tube is passed posteriorly between the neck of pancreas and portal vein to facilitate dissection.

She underwent an open central pancreatectomy (Figure 2). The proximal stump of the pancreas was managed by suture closure reinforced with a falciform patch and the distal pancreatic stump was managed by PG by modified binding technique.

In the postoperative period, she had chylous ascites (Clavien dindo Grade 1) managed with a high protein, low-fat diet, and diuretics and delayed gastric emptying (ISGPS Grade A) which was managed with prokinetic drugs. The length of the hospital stay was 10days. At one year follow-up, she did not develop glucose intolerance or symptoms of exocrine insufficiency. There was no recurrence of the tumor.

## Discussion

Central pancreatectomy by definition is a form of segmental pancreatic resection which involves removal of neck and variable extent of the body of pancreas. On the basis of contiguous vascular structures, the pancreas is divided into head, body, and tail for localizing tumors according to American Joint Committee on Cancer eighth edition. The head of pancreas is the part of the gland situated to the right of the superior mesenteric-portal vein confluence. The body of the pancreas is the region between the left border of the superior mesenteric vein and the left border of the aorta. The tail of the pancreas lies between the left border of the aorta and splenic hilum [6]. The neck is the anteroposteriorly flattened portion of the head of the pancreas, anterior to the portal vein and medially grooved by the gastroduodenal artery. The neck is considered an integral part of the head of the pancreas and since the discrete definition does not add to the clinical value, it was possibly omitted deliberately. The procedure essentially involves the following steps - a division of gastrocolic omentum, exposure of pancreas, dissection of the pancreas from the portosplenomesenteric confluence which may include ligation of multiple venous tributaries draining into the splanchnic veins, pancreatic division, and reconstruction.

Although initially applied for malignant tumors, central pancreatectomy has fallen out of favor in malignancies due to the risk of positive margins. Head cancers and cancers of the body and tail both have shown intrapancreatic spread. There are two types of intrapancreatic spread - interstitial spread and intraductal spread: either one can be predominant. Studies by Yamamura et al. have shown that a 2-cm margin may not be adequate. Frozen sections are useful in determining the margin positivity but it is always better to avoid a margin positive transection to avoid spillage of tumor cells. The need for wider margins thus limits the role of central pancreatectomy in malignancies. Also, there is a theoretical risk of residual disease at lymph node stations such as splenic hilum and retro-pancreatic region which further diminishes the applicability [7].

Benign and borderline malignant tumors of the neck and proximal body of the pancreas, which include cystic tumors of the pancreas such as SCN, MCN, IPMN, SPT, pancreatic neuroendocrine neoplasms, and rarely abdominal trauma involving pancreatic body or neck are the current indications [8,9].

Our primary focus will be on the reconstructive methods following a central pancreatectomy.

Proximal stump

Reconstruction following central pancreatectomy assumes greater significance as there are two pancreatic stumps to deal with. Stapled closure, hand-sewn closure, hand-sewn over stapler closure, stapled closure with reinforcements such as vascularized falciform patch, omental patch, seromuscular patch, fibrin glue, surface-active mesh are the possible strategies described in the literature with almost similar morbidity. These results are extrapolated predominantly from distal pancreatic resections and the type of closure remains the surgeon’s choice based on their mentoring and experience. In a meta-analysis of 80 studies published on pancreatic stump closure done by Tieftrunk et al., a combination of stapler and suture closure of the main pancreatic duct demonstrated slightly lower fistula rates than any of the methods used alone.

We preferred hand-sewn closure wherein individual duct is closed with a purse-string first and then trans-pancreatic U sutures avoiding the duct were taken with a vascularized falciform patch as on-lay taking cues from Blumgart technique of PJ anastomosis. Thereby, sandwiching the pancreas in between the falciform may result in fewer leaks from accessory pancreatic ducts [10].

Distal stump

Management of the distal stump attracts more attention as the debate between PG and pancreatojejunostomy (PJ) steps in. Application of the results of reconstructive techniques during conventional pancreaticoduodenectomy to central pancreatectomy may be naive. The reason being, distal division of the body of the pancreas is required during a central pancreatectomy. In the setting of central pancreatectomy, PG has an unambiguous advantage over PJ in that it does not require discontinuity of the small bowel [11].

Pancreaticojejunostomy

Although PG is theoretically safer and technically less demanding, PJ is the most commonly performed pancreaticoenteric anastomosis worldwide owing to standardization of surgical training and regional bias indicated by the fact that roughly 3% of North American surgeons prefer PG vs 16% in other parts of the world [12]. In spite of extensive studies trying to prove the superiority of one technique over the other, to date, there has been no consensus on the same [13].

 The PJ anastomosis can be performed in an end-to-end or end-to-side fashion using a Roux limb of the jejunum. It can be invaginating/dunking PJ, binding PJ using single or double-layer purse-string sutures, or duct-to-mucosa technique [14]. Another method described is pancreatic duct eversion or evagination to maintain its patency [15]. Trans-anastomotic stents, fibrin glues, and omental wrapping in PJ have been reported but with no added benefit.

Pancreaticogastrostomy

Several techniques of performing a PG exist. The commonly performed ones are the duct-to-mucosa technique, invagination of the pancreatic stump into the gastric lumen, and binding PG. The use of transanastomotic stents for drainage of pancreatic secretions has been described in the literature as has been the use of fibrin glues and omental wrapping, but its use is limited. Further modifications described in the literature include duct-to-mucosa PG with single layer transfixing sutures between posterior gastric wall and pancreatic remnant, binding PG with two-layer purse-string sutures, and fashioning a PG with end-to-side duct-to-mucosa anastomosis by the creation of gastric partition [16]. All the methods usually require variable mobilization of the remnant pancreas to ensure a tension-free anastomosis.

We preferred a binding PG using a single layer purse-string sutures along with trans pancreatic U sutures to prevent leakage from minor pancreatic ducts and cut the surface of the pancreas. The anastomosis was preceded by mobilization of the distal pancreatic stump up to three centimeters.

Mobilization and relevance of vascular anatomy to extent of mobilization

Understanding the vascularity of the body and tail of the pancreas is paramount when one is planning a distal pancreatic reconstruction. It can be divided into superior & inferior bodies supplied by the dorsal pancreatic artery & greater pancreatic artery respectively, superior tail & tail end by caudal pancreatic arteries, and the inferior tail by transverse pancreatic and greater pancreatic arteries. There is great variability in the arterialization of the distal pancreas. Studies done on cadaveric pancreatic specimens and live patients using CT Angiography of the celiac axis have thrown more light on the vasculature of the pancreas. Roman Ramos et al. while studying the vasculature of the body and tail of the pancreas listed four main patterns of blood supply, all derived from the splenic artery: type I: small arcades, type II: small and large arcades, type III: large arcades only, type IV: straight branches. Amongst these, types I & II are the most common patterns of vascularization [17]. The pattern of vascularity may have effects on the viability of the pancreatic stump after mobilization. In type IV patterns, excessive mobilization may lead to anastomotic failure owing to the segmental pattern of vascularity. With more insight into these patterns with the advent of advanced imaging, the extent of mobilization may be individualized. Most authors suggest mobilization of at least two to 4 cm [18]. However, when extensively mobilizing the distal pancreatic stump, one has to keep in mind the fragile vascularity of the body and tail of the pancreas so as to prevent vascular insufficiency which in turn will lead to anastomotic leak [19]. In trying to redefine the extent of mobilization, given the understanding, it would be to such an extent that it is just enough to adequately invaginate the pancreatic stump and not merely numbers. Also, the cross-sectional configuration of the pancreas changes triangular as we skew toward the tail. This combined with the ischemic effects of mobilization deserves further attention in reconstructive methods. Moreover, the differences in the extent of mobilization between PJ and PG are less well studied.

Octreotide and its effects in relation to mobilization

The use of octreotide in central pancreatectomy raises more controversy given the variable vascular pattern of the distal stump and the extent of mobilization. Due to its splanchnic vasoconstrictive action, octreotide can attenuate the blood supply further and promote a pancreatic fistula formation by impairing the healing process at the anastomotic site. Several authors have studied the effect of octreotide on rates of pancreatic fistula; few studies have shown any actual benefit and most authors would not recommend the routine pre or peri-operative use of octreotide in pancreatic surgery [20].

## Conclusions

Central pancreatectomy as a parenchyma sparing procedure has its own merits and demerits. The risk of increased morbidity must be weighed against the benefit of the preservation of exocrine and endocrine function. Further understanding of the vascular pattern of the pancreas by advancements in imaging modalities may help in individualizing the choice of reconstruction.
